# Endogenous insulin secretion and suppression during and after sepsis in critically ill patients: implications for tight glycemic control protocols

**DOI:** 10.1186/cc9809

**Published:** 2011-03-11

**Authors:** CG Pretty, PD Docherty, J Lin, L Pfeifer, U Jamaludin, GM Shaw, AJ Le Compte, JG Chase

**Affiliations:** 1 University of Canterbury, Christchurch, New Zealand; 2 University of Otago, Christchurch, New Zealand; 3 Christchurch Hospital, Christchurch, New Zealand

## Introduction

Insulin infusions over 2 U/hour can suppress endogenous insulin secretion in healthy subjects 30 to 45% [1]. Virtually all tight glycemic control (TGC) protocols deliver insulin via infusion. This study examines the impact of bolus delivery of insulin in TGC on the endogenous insulin secretion of critically ill patients.

## Methods

Eighteen patients from the Christchurch Hospital ICU enrolled in a prospective clinical trial studying sepsis each had two sets of blood samples assayed for insulin and C-peptide. The first set was taken at the commencement of the SPRINT TGC protocol for patients with suspected sepsis. The second set was taken when their SIRS score was consistently below 2. Each set had four samples taken at: -1, 10, 40 and 60 minutes following bolus delivery of insulin as required by SPRINT to capture endogenous insulin secretion during the bolus profile. Bolus size was dictated by the protocol, but was in the range 2 to 6 units. Model-based methods [2] were used to calculate the endogenous insulin secretion rate for each set of samples. The level of suppression was calculated as the ratio of the secretion rate between 5 and 15 minutes (just after peak plasma insulin) and average of the 0 to 5 minutes (basal) and 15 to 60 minutes (return to basal) secretion rates identified.

## Results

Median (IQR) endogenous insulin secretion rates for the first and second set of samples, respectively, were 4.0 (1.4 to 5.4) U/hour and 1.5 (1.0 to 3.3) U/hour, indicating a significant drop in secretion, post-sepsis and later in stay (*P *< 0.05). Median (IQR) level of suppression for the first set of samples of each patient was 1.08 (0.96 to 1.29), showing an increase in secretion for most patients during suspected sepsis. Second set suppression post-sepsis was 1.02 (0.83 to 1.12), indicating limited or no suppression outside C-peptide assay error of 9%. Analyses of blood glucose levels, culture-confirmed sepsis and diabetic status show no consistent trends.

## Conclusions

TGC can be beneficial, but carries a high risk of hypoglycemia. Bolus insulin may provide more effective TGC as unsuppressed endogenous insulin supplements the exogenous dose, possibly lowering the required doses and the risk of hypoglycemia. These results suggest a comparative study between bolus and infused insulin in TGC.
